# Variation in the Number and Position of rDNA Loci Contributes to the Diversification and Speciation in *Nigella* (Ranunculaceae)

**DOI:** 10.3389/fpls.2022.917310

**Published:** 2022-06-09

**Authors:** Fatemeh Orooji, Ghader Mirzaghaderi, Yi-Tzu Kuo, Jörg Fuchs

**Affiliations:** ^1^Department of Agronomy and Plant Breeding, Faculty of Agriculture, University of Kurdistan, Sanandaj, Iran; ^2^Leibniz Institute of Plant Genetics and Crop Plant Research (IPK), Gatersleben, Germany

**Keywords:** repetitive sequences, satellites, karyotype evolution, *Nigella* genus, repeatome analysis

## Abstract

*Nigella* is a small genus belonging to the Ranunculaceae family which is presumably originated and distributed in Aegean and the adjacent Western-Irano-Turanian region. Comparative repeat analysis of *N. sativa*, *N. damascena* and *N. bucharica* was performed using low-pass Illumina genomic reads followed by karyotyping and FISH mapping of seven *Nigella* species using the *in silico* identified repeats and ribosomal DNA (rDNA) probes. High- and moderate-copy repeat sequences occupy 57.52, 59.01, and 64.73% of *N. sativa*, *N. damascena* and *N. bucharica* genomes, respectively. Roughly, half of the genomes are retrotransposons (class I transposons), while DNA transposons (class II transposons) contributed to only about 2% of the genomes. The analyzed *Nigella* species possess large genomes of about 7.4 to 12.4 Gbp/1C. Only two satellite repeats in *N. sativa*, one in *N. damascena* and four in *N. bucharica* were identified, which were mostly (peri)centromeric and represented about 1% of each genome. A high variation in number and position of 45S rDNA loci were found among *Nigella* species. Interestingly, in *N. hispanica*, each chromosome revealed at least one 45S rDNA site and one of them occurs in hemizygous condition. Based on the chromosome numbers, genome size and (peri)centromeric satellites, three karyotype groups were observed: Two with 2*n* = 2*x* = 12 and a karyotype formula of 10m + 2t (including *N. sativa*, *N. arvensis*, *N. hispanica* as the first group and *N. damascena* and *N. orientalis* as the second group) and a more distant group with 2*n* = 2*x* = 14 and a karyotype formula of 8m + 2st + 4t (including *N. integrifolia* and *N. bucharica*). These karyotype groups agreed with the phylogenetic analysis using ITS and *rbc*L sequences. We conclude that variation in (peri)centromeric sequences, number and localization of rDNA sites as well as chromosome number (dysploidy) are involved in the diversification of the genus *Nigella*.

## Introduction

*Nigella* (fennel flower) is a small genus in the tribe *Nigelleae* (with 18 species) of the Ranunculaceae family (Zohary, 1983; Dönmez et al., 2021) (Supplementary Table 1), native to Southern Europe, North Africa, South Asia, Southwest Asia and Middle East (Tutin, 1964; Zohary, 1983; Raab-Straube et al., 2014) (Supplementary Figure 1). Fourteen species belong to *Nigella*, among which *N. sativa* L. (black cumin) is the most popular medical plant and additionally its seeds are used as spices. *N. damascena* L. and *N. arvensis* are annual ornamental and medicinal plants (Ghosh and Datta, 2006; Malhotra, 2012; Shaker et al., 2017). *Komaroffia bucharica* and *K. integrifolia* belong to the *Komaroffia* tribe (the sister tribe of *Nigelleae*), that Zohary accepted as synonyms for *Nigella bucharica* and *N. integrifolia* (Zohary, 1983; Heiss et al., 2011; Dönmez et al., 2021). *N. bucharica* and *N. integrifolia* are of great importance for beekeeping, as they provide bees with nectar and pollen in south Uzbekistan (Atamuratova et al., 2021). The diploid species, *N. sativa, N. damascena, N. arvensis, N. hispanica* and *N. orientalis* (2*n* = 2*x* = 12), have five metacentric and one telocentric chromosome pairs, but *N. bucharica* and *N. integrifolia* (2*n* = 2*x* = 14) have four metacentric, two submetacentric and one subtelocentric chromosome pairs (Gilot-Delhalle et al., 1976). The 1C-values of *N. sativa* and *N. damascena* were determined to be 10.39 Gbp (Bennett and Smith, 1976) and 10.29 Gbp (Evans et al., 1972; Kuznetsova et al., 2017; Leitch et al., 2019), respectively. There is little information about the genome composition and cytogenetic characteristics of *Nigella* species although such information is important to understand the phylogenetic relationship in this genus.

Repetitive DNAs are highly enriched in plant genomes, and repetitive fractions among plant genomes are highly variable, ranging for example from 13–14% in the small genome of *Arabidopsis thaliana* (157 Mbp/1C) (Bennett et al., 2003), to up to 92% in *Allium cepa* with a rather large genome (16 Gbp/1C) (Fu et al., 2019). Transposons and tandem repeat DNAs (including satellites and ribosomal DNAs) are major repetitive sequences in eukaryotic genomes (Wicker et al., 2007; Mehrotra and Goyal, 2014; Bao et al., 2015; Piégu et al., 2015; Maumus and Quesneville, 2016). Satellites are commonly used as molecular and cytogenetic markers in studies of the genetic diversity and chromosome evolution due to their species-, or even chromosome-specificity (Elder and Turner, 1995; Ugarkovic and Plohl, 2002; Garrido-Ramos, 2017; Samoluk et al., 2017; Belyayev et al., 2019). Although 45S (18S-5.8S-25S) and 5S rDNA have been widely used as cytological markers for chromosome identification and investigations of chromosomal rearrangements occurring between related species (Mukai et al., 1991; Zoldos et al., 1999; Frello and Heslop-Harrison, 2000; Tagashira and Kondo, 2001; Datson and Murray, 2003), the ITS sequences of 45S rDNA are rather variable between species. In addition to large-scale chromosomal rearrangements, such as inversions and translocations, the high variation in copy number and distribution of tandem repeats can lead to genome divergence and karyotype changes between closely related species (Appels et al., 1980; Mukai et al., 1991; Levin and Donald, 2002).

In this study, we analyzed and compared the repeat composition of *N. sativa*, *N. damascena* and *N. bucharica* using low-coverage genome sequences. Furthermore, we generated karyotypes of seven *Nigella* species using FISH mapping of major satellite repeats and rDNAs. Types and patterns of satellite repeats and number of chromosomes agreed with the phylogenetic relationships revealed by using ITS and *rbc*L sequences.

## Materials and Methods

### Plant Materials

Seeds of seven *Nigella* species, *N. sativa*, *N. damascena*, *N. arvensis*, *N. bucharica*, *N. hispanica*, *N. integrifolia* and *N. orientalis*, were provided by the IPK Genebank in Germany (Table 1, Supplementary Table 1, and Supplementary Figure 2). All species were used for phylogenetic analysis and FISH karyotyping. *N. sativa*, *N. damascena* and *N. bucharica* were further used in a comparative analysis of their genome repetitive compositions.

**TABLE 1:** Nigella species and accessions used in the present study.

*Nigella* species	Accession number^a^	Chromosome number (2*n* = 2*x*)	Genome size (Gb/1C)	Pair number of 45S rDNA loci	Pair number of 5S rDNA loci
*N. arvensis* L.	NIGE 5	12	7.851	4	1
*N. bucharica* Schipcz.	NIGE 15	14	7.398	3	1
*N. damascena* L.	NIGE 101	12	11.826	4	2
*N. hispanica* L.	NIGE 28	12	8.732	10	1
*N. integrifolia* Regel	NIGE 31	14	7.443	3	1
*N. orientalis* L.	NIGE 34	12	12.441	3	3
*N. sativa* L.	NIGE 61	12	11.719	3	2

*^a^Accession number of the Nigella species in IPK Genebank, Gatersleben, Germany.*

### Genome Size Measurement

To isolate nuclei, approximately 0.5 cm^2^ of fresh leaf tissue from a *Nigella* species and the internal reference standard, *Pisum sativum* L. subsp. *sativum* convar. *sativum* var. ponderosum Alef., Sorte Viktoria, Kifejtö Borsó, Gatersleben Gene Bank accession number: PIS 630, were chopped together in a petri dish using the reagent kit ‘CyStain PI Absolute P’ (Sysmex-Partec) following the manufacturer’s instructions. The nuclei suspension was filtered through a 50-μm CellTrics filter (Sysmex-Partec) and measured on a CyFlow Space flow cytometer (Partec-Sysmex). For each genotype, at least six independent measurements were performed. The absolute DNA content (pg/2C) was calculated based on the values of the G1 peak means and converted to the corresponding genome size (Mbp/1C) according to Dolezel et al. (2003).

### DNA Extraction and Sequencing

Genomic DNAs were extracted from the leaves of *N. sativa*, *N. damascena* and *N. bucharica* using the CTAB method described in Saghai-Maroof et al. (1984), Aboul-Maaty and Oraby (2019). Paired-end (2 × 150 bp) genome sequencing was performed using the Illumina HiSeq 2500 system in a low-coverage scale by Novogene (China). The coverage of sequenced genome was calculated according to the following equation: Coverage = (Number of reads × size of each read)/1C content of the genome.

### Graph-Based Identification of Genome Repetitive Sequences

The quality and GC content of paired-end reads of each species was checked using FastQC (Andrews, 2010) implanted in the RepeatExplorer. The sequence reads were filtered by the quality of 95% of bases equal to or above the quality cut of value of 10. Paired reads were joint using FASTA interlacer tool and pairs with no overlap were selected for the graph-based clustering analysis. The identification and characterization of the repetitive DNA families were then performed using the RepeatExplorer pipeline (Novak et al., 2013; Novák et al., 2017, 2020) with the default setting of 90% similarity over 55% of the read length. Consensus sequences of the identified repeat monomers were reconstructed by TAREAN (TAndem REpeat ANalyzer) (Novák et al., 2017). Comparative RepeatExplorer analysis was performed to identify shared and species-specific repeat clusters. The “*Nd*,” “*Ns*,” and “*Nb*” were used as prefix codes of *N. damascena*, *N. sativa* and *N. bucharica*, respectively. The sequence dataset of each species was then down-sampled to 20% of each genome size (16 million reads for *N. sativa* and *N. damascena* and 10 million reads for *N. bucharica*), followed by a concatenation into a single data file. The settings for comparative clustering analysis were the same as those for individual analysis mentioned above. The sizes of repeat clusters were normalized based on the genome size of analyzed species using optparse package of R version 4.0.2 (The R Project for Statistical Computing, Vienna, Austria).

The monomer of (peri)centromeric satellite sequences were aligned using Clustal Omega (Madeira et al., 2019) and viewed in MView (Brown et al., 1998). The 18S, 5.8S, and 26S coding regions of the identified 45S rDNA and the coding region of 5S rDNA were distinguished by referring to the publicly available rDNA coding sequences in NCBI.

### Polymerase Chain Reaction

To amplify the repeat DNAs, the consensus sequences of satellite repeats and one LTR element identified were used to design primers using Primer3 (Untergasser et al., 2012). The monomer and primer sequences were listed in Supplementary Table 2. The PCR mixture contained 25 ng of genomic DNA as template, 2.5 mM of each dNTP, 2.5 mM MgCl_2_, 5 pmol of each primer, and 0.5 U *Taq* DNA polymerase. PCR amplification was performed for 5 min at 94°C, followed by 30 cycles of 30 s at 94°C, 1 min at 52-60°C (depending on primers), 1 min at 72°C and a final extension for 7 min at 72°C. The size of PCR products was checked in 1% agarose gel by electrophoresis.

### Probe Preparation

PCR products were purified by ethanol precipitation. One microgram of each purified PCR products was labeled with Atto488-11-dUTP or Atto550-11-dUTP using a nick translation kit (Jena Bioscience, Germany), recovered by ethanol precipitation and used as FISH probes. For rDNA probes, the 45S rDNA and 5S rDNA containing clones p*Ta*71 (Gerlach and Bedbrook, 1979) and p*Ta*794 (Gerlach and Dyer, 1980), respectively, were labeled with Atto488-11-dUTP and Atto550-11-dUTP by nick translation as mentioned above. To investigate whether *Nigella* species possess Arabidopsis-like telomeric repeats, FISH was performed using Arabidopsis-type telomere repeats (TTTAGGG)_n_ as a probe, which was generated by non-template PCR according to IJdo et al. (1991) using (TTTAGGG)_3_ and (CCCTAAA)_3_ as primers. One microgram of the purified PCR product was labeled with Atto550-11-dUTP as described above, recovered by ethanol precipitation and used as FISH probes.

### Slide Preparation

*Nigella* seeds were germinated on moist filter paper in petri dishes for 3–6 days at room temperature. Roots were subjected to nitrous oxide (N_2_O) gas at 10 bar pressure for 2 h to arrest dividing cells at metaphase. Treated roots were fixed in ice-cold 90% acetic acid for 10 min, then transferred to 75% ethanol and stored at −20°C until use. Roots were first washed in ice-cold water, followed by 0.01 M citrate buffer (0.01 M citric acid and 0.01 M sodium citrate, pH 4.8) each for 10 minutes. Root meristems were placed in a microtube containing 30 μl enzyme mixture [0.7% cellulase (CalBiochem 219466), 0.7% cellulase R10 (Duchefa C8001), 1% cytohelicase (Sigma C8274) and 1% pectolyase (Sigma P3026) in 0.01 M citrate buffer] and were digested at 37°C for 60 to 90 minutes. Slides were prepared using the dropping method according to Abdolmalaki et al. (2019). The specimens were fixed in 4% paraformaldehyde in 1 × PBS (3 mM NaH_2_PO_4_, 7 mM Na_2_HPO_4_, 0.13 M NaCl, pH 7.4) for 10 min at room temperature, followed by washing in 2 × SSC (0.3 M sodium chloride, 0.03 M sodium citrate, pH 7.0) and dehydrating in 96% ethanol.

### Fluorescence *in situ* Hybridization

FISH and reprobing was performed according to Abdolmalaki et al. (2019). Briefly, 20 μl of hybridization mixture, containing 2 × SSC, 50% formamide, 20% dextran sulfate, 1 μg sheared salmon testes DNA and 20–30 ng of each labeled probe, was applied on each slide and covered with a plastic coverslip. Specimens were then denatured at 80°C for 2 min on a hot plate and were incubated in a humidified plastic container at 37°C, overnight. Coverslips were removed and slides were washed in 2 × SSC for 20 minutes in a water bath at 56°C. Slides were dehydrated in 96% ethanol and dried at room temperature. A drop of Vectashield mounting medium (Vector Laboratories) containing 1 μg/ml DAPI (4′, 6-diamidino-2-phenylindole) was added to each slide as counterstain and a glass coverslip was applied. Slides were inspected with a fluorescence Olympus BX51 microscope (Olympus, Japan), and images were captured using a DP72 digital camera (Olympus, Japan).

### Numerical Characterization of Karyotypes

Chromosomal and karyotypic indices for numerical characterization of mitotic metaphase chromosomes of the *Nigella* species were measured using IdeoKar software (Mahmoudi and Mirzaghaderi, 2021). The calculated indices include total chromosome length of the haploid complement (HCL); mean chromosome length (CL), and mean centromeric index (CI). Karyotype asymmetry was determined using the A1 (intrachromosomal asymmetry index) and A2 (interchromosomal asymmetry index) indices calculated using Σ(b/B)/n and s/x equations, respectively, where b and B are the mean lengths of the short and long arms of each homologous chromosome pair, respectively; n is the number of homologs, and s and x are standard deviation and mean of the chromosome length, respectively (Romero-Zarco, 1986). Three high-quality FISH-banded metaphase chromosome spreads were traced for each species. The description of chromosome morphology was based on the nomenclature proposed by Levan et al. (1964). Idiograms were generated using the R package “idiogramFISH” (Roa and Telles, 2020).

### Phylogenetic Analysis

The ITS1-5.8S-ITS2 region of 45S rDNA in *N. sativa*, *N. damascena* and *N. bucharica* were identified by RepeatExplorer analysis and were extracted using BLASTn at NCBI database. *N. damascen*a complete chloroplast sequence (MN648403.1) was downloaded from NCBI and used as a reference genome to assemble the chloroplast sequence of *N. sativa* using CLC software. The reference-aided assembled genome was annotated using the GeSeq annotation tool (Tillich et al., 2017). The conserved sequences flanking the ITS1-5.8S-ITS2 and *rbc*L gene were used to design PCR primers to amplify and sequence the corresponding regions in the other five *Nigella* genomes (Supplementary Tables 2–4). The ITS1-5.8S-ITS2 and *rbc*L sequences were used as input for multiple sequence alignment by MUSCLE algorithm using MEGA11 software (Tamura et al., 2021). The concatenated ITS1-5.8S-ITS2 and *rbc*L sequences were used to build a maximum likelihood tree with 500 bootstrapping replications in MEGA11.

## Results

### *Nigella* Is Characterized by Relatively Large Genomes

**FIGURE 1:**
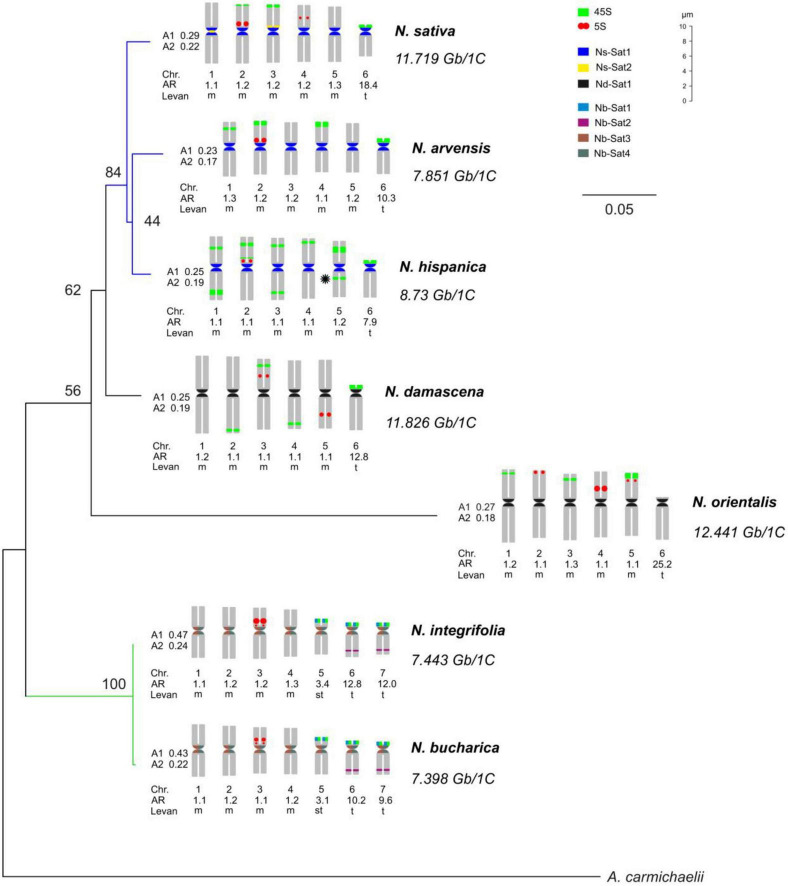
Phylogeny and idiograms of *Nigella*. A maximum likelihood phylogenetic tree of *Nigella* species with 500 bootstrap replications inferred from ITS1-5.8S-ITS2 and *rbc*L sequences. A. carmichaelii has been included as an outgroup. The tree has been annotated with idiograms (showing the locations of the identified satellite repeats and 45S and 5S arrays), karyotypic parameters and the estimated DNA C-values. The asterisk in *N. hispanica* indicates the hemizygous locus. Chr.: Chromosome; A1: intrachromosomal asymmetry index; and A2: interchromosomal asymmetry index; Levan: the description of chromosome morphology was based on the nomenclature proposed by Levan et al. (1964), AR: arm ratio (long arm/short arm).

According to flow cytometric estimation of the DNA content, *Nigella orientalis* has the largest genome with 12.44 Gbp/1C among the seven species, followed by *N. damascena* and *N. sativa* with 11.72 Gbp/1C and 11.83 Gbp/1C, respectively (Table 1 and Figure 1). *N. hispanica* (8732 Mbp/1C), *N. arvensis* (7851 Mbp/1C), *N. integrifolia* (7443 Mbp/1C) and *N. bucharica* (7398 Mbp/1C) have considerably smaller genomes than those of the other three species mentioned above.

### Two Different Karyotypes Are Prevailing in *Nigella*

Chromosomes of *Nigella* species were mainly metacentric with one or two telocentric chromosome pairs in each species. Based on their basic chromosome number, the seven species can be classified into two groups. The first group, comprising *N. arvensis*, *N. damascena*, *N. hispanica*, *N. orientalis* and *N. sativa*, has a basic chromosome number of *x* = 6 (2*n* = 2*x* = 12) with a karyotype formula of 10m + 2t. *N. bucharica* and *N. integrifolia* belong to the second group with a basic chromosome number of *x* = 7 (2*n* = 2*x* = 14) and a karyotype formula of 8m + 2st + 4t. All these species fell into the 2A category of Stebbin’s asymmetry indices (Stebbins, 1971; Figure 1). The size of metacentric chromosomes ranged from 5.99 μm (*N. bucharica*) to 10.14 μm (*N. damascena*) and the telocentric chromosome size ranged from 4.04 μm (*N. bucharica*) to 5.51 μm (*N. damascena*). *N. bucharica* and *N. integrifolia* also have a pair of subtelocentric chromosomes with a size range from 4.54 to 4.71 μm. The total metaphase chromosome length was between 38.21 μm in *N. bucharica* and 53.17 μm in *N. damascena* (Supplementary Table 5).

### The Number of rDNA Loci Varies Severely Between the Species

**FIGURE 2:**
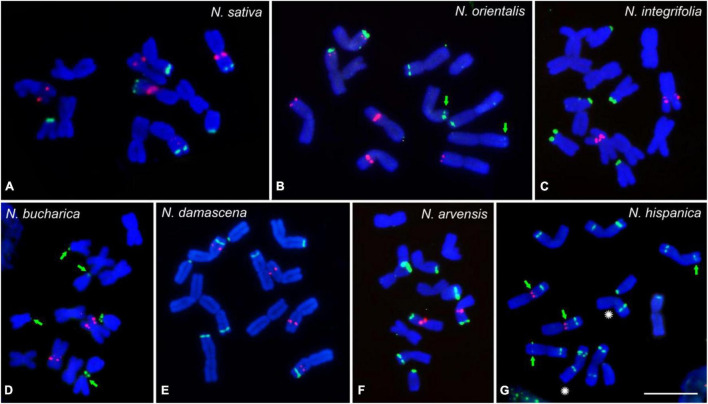
rDNA loci distribution of the studied Nigella species. FISH mapping of 45S rDNA (green) and 5S rDNA (red) on mitotic metaphase chromosomes of *N. sativa* **(A)**, *N. orientalis* **(B)**, *N. integrifolia* **(C)**, *N. bucharica* **(D)**, *N. damascena* **(E)**, *N. arvensis* **(F)** and *N. hispanica* **(G)**. Chromosomes were counterstained with DAPI. Arrows indicate weak 45S rDNA signals and the asterisks indicate the homologous chromosomes with hemizygous locus. Scale bar 10 μm.

To determine the karyotype evolution among the seven *Nigella* species, FISH mapping of 45S and 5S rDNA loci on mitotic chromosomes was performed (Figure 2). FISH of both ribosomal probes revealed a considerable interspecific variation regarding the number and position of rDNA loci (Figures 1A, 2). While three 45S rDNA-positive chromosome pairs were observed in *N. sativa*, *N. orientalis*, *N. integrifolia* and *N. bucharica* (Figures 2A–D), four pairs of 45S rDNA loci were present in *N. damascena* and *N. arvensis* (Figures 2E,F). *N. hispanica* revealed ten pairs of 45S rDNA loci, the highest number among the investigated species. Each chromosome of this species harbors at least one 45S rDNA locus. Interestingly, one of the 45S rDNA sites in *N. hispanica* did not show a signal on its corresponding homologous chromosome representing hemizygosity (Figures 1A, 2G). While in *N. sativa*, *N. arvensis, N. hispanica* and *N. damascena*, 45S rDNA loci were found on metacentric and telocentric chromosomes, 45S rDNA loci were exclusively found on metacentric chromosomes in *N. orientalis* or on submetacentric and telocentric chromosomes in *N. bucharica* and *N. integrifolia* (Figure 2). The 5S rDNA was found on one (*N. integrifolia*, *N. bucharica*, *N. arvensis* and *N. hispanica*) (Figures 2C,D,F,G), two (*N. sativa* and *N. damascena*) (Figures 2A,E) or three (*N. orientalis*) (Figure 2B) chromosome pairs. 45S rDNA loci are located mainly either in distal or proximal regions of the chromosome arms, while 5S rDNA arrays were also found interstitially. The size of hybridization signals varied between chromosome pairs both within and between species (Figure 2).

### Molecular Phylogenetic Analysis of ITS and *rbc*L Sequences Correlate With the Basic Chromosome Numbers

To determine the phylogenetic relationship among the analyzed *Nigella* species, the sequences of nuclear ribosomal internal transcribed spacer (ITS) and *rbc*L gene were used. The sequence length of ITS (ITS1-5.8S-ITS2) varied from 732 to 759 bp (Supplementary Table 3), whereas *rbc*L sequences ranged from 871 to 1428 bp (Supplementary Table 4) in the seven *Nigella* species. *Aconitum carmichaelii*, a distantly related species belonging to the same family, was used as an outgroup, and the resulting consensus had high bootstrap support values (Figure 1). *N. bucharica* and *N. integrifolia* formed a robust cluster with 100% bootstrap support, and both of them have a basic chromosome number of *x* = 7. The other cluster included *N. sativa*, *N. arvensis* and *N. hispanica* (with 84% bootstrap support), to which *N. damascena* and *N. orientalis* were jointed with lower support. All members of this cluster possess a chromosome number of 2*n* = 12.

The clustering of the seven *Nigella* species based on molecular phylogenetic analysis correlates with their basic chromosome number (*x* = 6 or 7). The phylogenetically close *N. integrifolia* and *N. bucharica* have the same chromosome number, similar genome size and rDNA-based karyotypes. Nevertheless, among the other five species, despite having the same chromosome number, their genome size, the number and chromosomal distribution of rDNA loci are diverse.

### Retroelements Are the Dominating Repeat Type in *Nigella* While Satellite Sequences Are Rare

Low-pass sequencing of *N. sativa*, *N. damascena* and *N. bucharica* genomes resulted in 4,232,251, 7,553,644, and 15,352,348 Illumina 150 bp paired-end reads corresponding to 0.24×, 0.43×, and 1.50× genome coverage, respectively. The GC content for *N. sativa* and *N. damascena* genomes showed a value of 38%, while this value was 42% for *N. bucharica*. The repeat compositions were inferred from the paired-end reads corresponding to approximately ∼0.2× of the genome for each analyzed species. The proportions of individual repeat types are presented in Table 2. About 57.52, 59.01, and 64.73% of *N. sativa*, *N. damascena* and *N. bucharica* genomes are composed of high- or moderate-copy repeats, respectively. The majority of the repeats (47.91% in *N. sativa*, 39.47% in *N. damascena*, and 51.25% in *N. bucharica*) are retroelements, followed by unclassified repeats (6.95, 17.30, and 10.10%) and tandem repeats (0.75, 0.39, and 0.74% of rDNAs and 0.75, 1.21, and 1.45% of satellites). The proportions of 45S rDNA repeats in *N. sativa*, *N. damascena* and *N. bucharica* genomes were 0.72, 0.38, and 0.65%, respectively, while the 5S rDNA proportions were 0.03%, 0.01% and 0.09% as determined by RepeatExplorer analysis (Table 2). The consensus monomers of the rDNA sequences in *N. sativa*, *N. damascena* and *N. bucharica* identified by TAREAN are listed in Supplementary Table 6. Among the retroelements, LTR retroelements are the most abundant in the *N. sativa* (47.91%), *N. damascena* (39.47%) and *N. bucharica* (51.25%) genomes. LTRs in *N. sativa* include Ty3–gypsy and Ty1–copia super families with a proportion of 44.01 and 3.76% in the genome, respectively, while they compose 37.77 and 1.64% in *N. damascena* and 48.55 and 2.58% in *N. bucharica*. A major part (34.89% in *N. sativa*, 26.19% in *N. damascena* and 30.99% in *N. bucharica*) of Ty3–gypsy belongs to the retrotransposon chromoviral Tekay clade (Table 2). In contrast, DNA transposons contribute to only 1.13, 0.62, and 1.19% of the *N. sativa*, *N. damascena* and *N. bucharica* genomes, respectively, and only three common DNA transposons, EnSpm_CACTA, MuDR_Mutator and PIF_Harbinger, were identified. EnSpm_CACTA composes 0.72% of the *N. sativa* genome, but its proportion was much lower in *N. damascena* (0.24%) and *N. bucharica* (0.16%). MuDR_Mutator comprises about 0.32% of *N. sativa*, 0.34% of *N. damascena* and 0.71% of *N. bucharica* genome. Also, PIF_Harbinger composes 0.32% of the *N. bucharica* genome, but its proportion was lower than 0.1% in *N. sativa* (0.06%) and *N. damascena* (0.04%). The DNA transposon hAT was only detected in *N. sativa* (Table 2).

**TABLE 2:** Types and proportions of highly-repetitive sequences in *N. sativa*, *N. damascena* and *N. bucharica* characterized by RepeatExplorer2.

Repeat			Genome proportion (%)		
			*N. sativa*	*N. damascena*	*N. bucharica*
LTR retroelement	Ty1_copia	Angela	2.47	0.61	1.11
LTR retroelement	Ty1_copia	Bianca	0.14	0.08	0.02
LTR retroelement	Ty1_copia	Ikeros	0.21	0.12	0.13
LTR retroelement	Ty1_copia	Ivana	0.11	0.06	0.02
LTR retroelement	Ty1_copia	SIRE	0.45	0.39	−
LTR retroelement	Ty1_copia	TAR	0.24	0.23	0.77
LTR retroelement	Ty1_copia	Tork	0.13	0.14	0.53
LTR retroelement	Ty1_copia	Ale	0.01	0.01	−
LTR retroelement	Ty3_gypsy	Athila	6.46	8.26	7.23
LTR retroelement	Ty3_gypsy	Retand	2.33	2.65	10.04
LTR retroelement	Ty3_gypsy (chromovirus)	CRM	0.31	0.55	0.26
LTR retroelement	Ty3_gypsy (chromovirus)	Tekay	34.89	26.19	30.99
LTR retroelement	Ty3_gypsy (chromovirus)	Galadriel	0.02	0.09	0.03
LTR retroelement	Ty3_gypsy (chromovirus)	Reina	−	0.03	−
LTR retroelement	LINE		0.14	0.06	0.12
Total LTR retroelement			47.91	39.47	51.25
Pararetrovirus			0.03	0.02	−
DNA transposon		EnSpm_CACTA	0.72	0.24	0.16
DNA transposon		MuDR_Mutator	0.32	0.34	0.71
DNA transposon		hAT	0.03	−	−
DNA transposon		PIF_Harbinger	0.06	0.04	0.32
Total DNA transposon			1.13	0.62	1.19
rDNA		45S_rDNA	0.72	0.38	0.65
rDNA		5S_rDNA	0.03	0.01	0.09
Satellite			0.75	1.21	1.45
Tandem repeads			1.50	1.60	2.19
Unclassified			6.95	17.30	10.10
Total high- or moderate copy repeats			57,52	59,01	64,73
Non-clustered reads (low-copy sequences)			42.48	40.99	35.27

*The repeats grouped according to their repeat class and lineage. “-”: not detected.*

**FIGURE 3:**
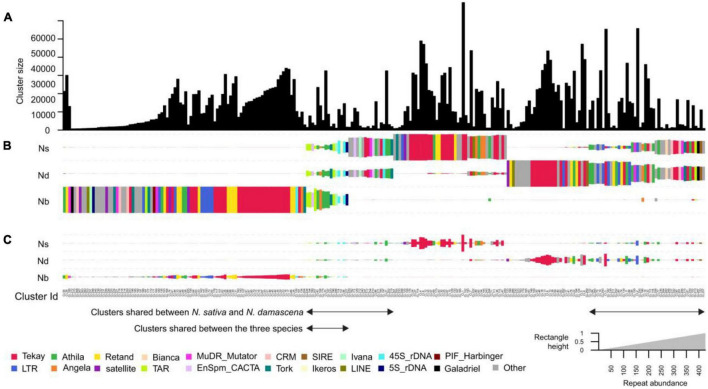
Comparative analysis of genome repetitive composition among three Nigella species. **(A)** Bar plot of N. damascena, N. sativa and N. bucharica showing the sizes (numbers of reads) of individual clusters. **(B)** Rectangle size is proportional to the number of reads in a cluster for each species. Clusters were sorted by using hierarchical clustering and the rectangles are colored based on their cluster annotation. **(C)** The size of the rectangles in **(B)** was normalized based on the genome size of analyzed species. Species codes: ND, *N. damascena*; NS, *N. sativa*; Nb, *N. bucharica*.

To compare the repeat compositions between the genomes of *N. sativa*, *N. damascena* and *N. bucharica*, a comparative clustering analysis was performed. About a quarter of the top clusters (Figure 3) are shared between the species. Not all of these clusters had similar abundance in the genomes. Out of the in total 272 major repeat clusters, only 16 clusters (5.88%) were relatively evenly shared between the three genomes, and they were annotated as Ty1_copia-TAR and Tork, Ty3_gypsy-Athila, DNA transposon-EnSpm CACTA and rDNAs (Figure 3). Up to 97 clusters (35.66%) were almost *N. bucharica* specific, and shared clusters between *N. bucharica* and either *N. damascena* or *N. sativa* were barely detectable. *N. damascena* and *N. sativa* contributed to 123 and 120 clusters, respectively, of which 77 clusters were shared between the two genomes, whereas 61 and 37 of them were highly enriched or specific to *N. damascena* and *N. sativa*, respectively. The comparative analysis demonstrated that *N. bucharica* is relatively more distinct from *N. damascena* and *N. sativa*. This result is in line with their phylogenetic relationships inferred based on ITS and *rbc*L sequences. The monomer length and cluster proportion of satellites and high copy retrotransposons identified by TAREAN is listed in Supplementary Table 7. Most of retrotransposons were common between *N. sativa* and *N. damascena*, (Supplementary Table 7).

### (Peri)centromeric Satellites Reflect the Phylogenetic Relationship in *Nigella*

The application of the TAREAN pipeline (Novák et al., 2017) allowed the identification of tandem repeat clusters in *Nigella* species. Two satellite repeats in *N. sativa*, i.e., Ns-Sat1 (CL21) and Ns-Sat2 (CL144) were detected, representing 0.52 and 0.01% of the genome, respectively (Table 3). Only one satellite repeat, named Nd-Sat1 (CL23), was identified in *N. damascena* which corresponds to 0.52% of the genome. On the other hand, four satellite repeats, Nb-Sat1 (CL21), Nb-Sat2 (CL129), Nb-Sat3 (CL64) and Nb-Sat4 (CL144), were identified in *N. bucharica*, representing 0.86, 0.08, 0.42, and 0.03% of the genome, respectively. All these repeats represented satellite-typical globular graph layouts, and their consensus monomer sequences are available in Supplementary Table 2. The monomers of Ns-Sat-1, Ns-Sat-2 and Nd-Sat-1 are all 178 bp in length and AT-rich (e.g., 68% AT for Ns-Sat1) (Table 3 and Supplementary Figure 3). Their sequence similarity ranged from 78.8% (between Ns-Sat1 and Nd-Sat1) to 71.8% (between Ns-Sat1 and Ns-Sat2). In addition, the monomer sequence of the Ty3_gypsy LTR-annotated retrotransposon, Ns-CL6, was reconstructed. To determine the chromosomal distribution of the identified repeats, the corresponding DNA fragments were PCR amplified using the respective primers and labeled as FISH probes (Table 3).

**TABLE 3:** Repeats used as probes in FISH experiments on *N. sativa*, *N. damascena* and *N. bucharica* chromosomes.

Repeat	Cluster	Repeat type	Monomer bp	Genome proportion%	PCR Primers (5′→ 3′)
Ns-Sat1	CL21	Satellite	178	0.52	F: AAGATCGCGTAAAACAGACGA R: TCAAAAACTTGAACGAATTCAAAA
Ns-Sat2	CL144	Satellite	178	0.013	F: ATCCGCTCGTTCGTCCATTT R: TCATTCGCGTAAAACTCGTGA
Ns-CL6	CL6	Transposon	6,465	2.3	F: AGGCAAACCAGGTACCACTG R: TTGGCAAATGGATGTCAAGA
Nd-Sat1	CL23	Satellite	178	0.52	F: CATGTAATGACAAACGGATCG R: TCAAAGGTTTGCTAATTTTCCA
Nb-Sat1	CL64	Satellite	21	0.42	F: TGGGGTTGGCAAGGCATG R: GGCCATGCCTTGCCAACC
Nb-Sat2	CL144	Satellite	159	0.034	F: GACAATTCGGGTCTTCGC R: GTTTCTTCACTATGGTCCCCC
Nb-Sat3	CL21	Satellite	135	0.86	F: TGAATTTGCAATAAACACCAAG R: CTTGCCATTTCATGACTTTCG
Nb-Sat4	CL129	Satellite	39	0.077	F: TTGCAAGTTCTTGAGTTTCT R: TGCAAGAAACTCAAGAACTT

*Repeat type, monomer length, their proportion in genome, and primer pairs used for their amplification are indicated. Other information including monomer sequences and FISH conditions are presented in Supplementary Table 3.*

**FIGURE 4:**
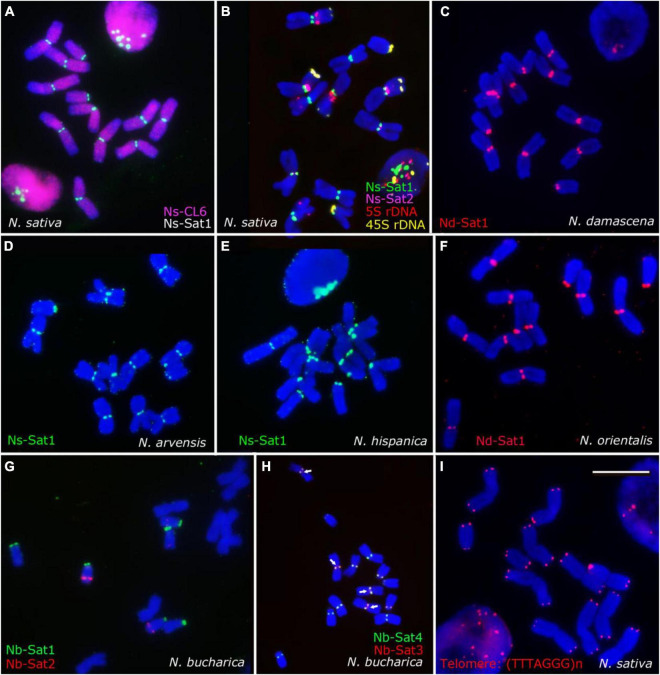
Chromosomal distribution of the identified high-copy repeats of Nigella. FISH results using the identified satellite repeats and Arabidopsis-type telomeric repeat probe (TTTAGGG)n probes on mitotic metaphase chromosomes of *N. sativa* **(A,B,I)**, *N. damascena* **(C)**, *N. arvensis* **(D)**, *N. hispanica* **(E)**, *N. orientalis* **(F)** and *N. bucharica* **(G,H)**. The Ns-CL6 (red) repetitive sequence is dispersed throughout the genome **(A)**, while the satellite sequences are mostly (peri)centromeric. Scale bar 10 μm.

After FISH, all metaphase chromosomes of *N. sativa* revealed (peri)centromeric Ns-Sat1 signals while Ns-Sat2 localized in the (peri)centromeric regions of only chromosomes 1 and 4 (Figures 4A,B). The Ns-CL6 probe which is a Ty3_gypsy LTR retrotransposon, resulted in evenly distributed signals, although with a lower density toward the distal chromosome regions (Figure 4A). Nd-Sat1-specific signals were found in the (peri)centromeric regions of all *N. damascena* chromosomes (Figure 4C). Ns-Sat1 also cross-hybridized to the (peri)centromeric regions of *N. arvensis* and *N. hispanica* (Figures 4D,E). The Nd-Sat1 of *N. damascena* also cross-hybridized to the (peri)centromeric regions of *N. orientalis* (Figure 4F). None of the Nd-Sat1, Ns-Sat1 and Ns-Sat2 probes cross-hybridized with *N. integrifolia* or *N. bucharica*. The observed clustering of (peri)centromeric repeats at one pole of the nuclei indicates a Rabl-like chromosome configuration in interphase nuclei of *Nigella* (Figures 4A,E).

The observed hybridization signals of all four Nb satellite probes showed similar intensities, locations and numbers in *N. bucharica* and *N. integrifolia* (Figure 1). Nb-Sat1 seems to co-localize with the 45S-rDNA loci, since it is found in terminal positions on the short arms of the two telocentric and the submetacentric chromosomes. Nb-Sat2 revealed signals on the distal ends of the long arm of the telocentric chromosomes (Figure 4G), while Nb-Sat3 and Nb-Sat4 showed signals in (peri) centromeric positions of all chromosomes (Figures 1A, 4H). It seems that at least in some of the chromosomes Nb-Sat3 is extended toward the inner part of the centomeres compared with Nb-Sat4 (arrows in Figure 4H). FISH with the *Arabidopsis*-type telomere repeat (TTTAGGG)n detected corresponding signals exclusively at both ends of all *Nigella* chromosomes (Figure 4I).

The genome-wide repetitive analysis in the three *Nigella* species indicated that retroelements, especially Ty_gypsy LTRs, are the main contributors to the relatively large genomes of *Nigella*. On the contrary, the abundance and diversity of satellite DNAs are relatively low. Most of these satellites locate at (peri)centromeric regions. The (peri)centromeric satellite repeats of *N. sativa* (Ns-Sat1), *N. damascena* (Nd-Sat1) and *N. bucharica* (Nb-Sat3 and Nb-Sat4) are highly distinct and cross-hybridized only to the closely related genomes as indicated in Figure 1.

## Discussion

We studied the phylogenetic relationship and karyotype structure of seven *Nigella* species by using sequences of ITS and *rbc*L gene, analyzing the repeatome and FISH mapping. Except for *N. sativa* and *N. damascena*, whose genome sizes were previously reported, the genome size of the other species was estimated for the first time. *N. orientalis*, *N. damascena*, and *N. sativa* have roughly 1.5 times larger genomes than *N. arvensis*, *N. hispanica, N. bucharica* and *N. integrifolia*. The DNA C-values estimated for *N. sativa* and *N. damascena* were quite similar to the previous estimations based on Feulgen densitometry [10.30 and 10.58 Gbp/1C for *N. damascena* (Evans et al., 1972; Olszewska and Osiecka, 1983) and 10.39 Gbp/1C for *N. sativa* (Bennett and Smith, 1976). The slight differences might be explained by the different methods used (Feulgen densitometry versus flow cytometry) and/or the different reference standards used (*P. sativum* versus *Allium cepa*).

In spite of the smaller genome sizes, *N. bucharica* and *N. integrifolia* have with a genome formula of 2*n* = 2*x* = 14 one additional chromosome pair more than the other species (basic chromosome number *x* = 6). A previous study using Giemsa C-banding on *Nigella* chromosomes (Gilot-Delhalle et al., 1976) suggested that the telocentric chromosomes originated from a centromeric fission event of a metacentric chromosome. Subsequent structural rearrangements might have formed the submetacentric chromosome in *N. bucharica* and *N. integrifolia* (Gilot-Delhalle et al., 1976).

The morphology of the studied species is rather similar except for *N. bucharica* and *N. integrifolia*, which have distinct flower and leaf morphology (Supplementary Figure 3). Both species, in turn, show substantial morphological, karyological and sequence similarities, which raises the question if they should be classified as varieties of a single species instead of two independent species.

*N. sativa*, *N. damascena*, *N. arvensis*, *N. hispanica* and *N. orientalis* showed similar karyotypes in terms of chromosome numbers and morphology, but the patterns of 5S and 45S rDNA loci differ between species, suggesting that the evolution of these species was accompanied by chromosomal segment rearrangements such as inversions, translocations and Robertsonian fission or mobility of rDNA loci without noticeably affecting the arm ratios. Similarly, in a cytogenetic survey of the Ranunculaceae, the number, location and intensity of rDNA signals varied between various species of *Pulsatilla* and *Anemone* genera. Most of the 45S rDNA loci in these genus are located at distal regions of the short arms of acrocentric chromosomes, while 5S rDNA loci don’t show preferential chromosomal positions. Such a rDNA mobility might be the result of homologous and non-homologous recombination mechanisms and retroelement-mediated rDNA transpositions (Mlinarec et al., 2006, 2012; Sramkó et al., 2019). Variation in the number and position of rDNA loci has also been reported among other species such as e.g., legumes (Abirached-Darmency et al., 2005). rDNA might be moved by transposition, as previously shown in *Allium* species and their hybrids (Schubert, 1984). In fact, rDNA sequences are conserved, but their chromosomal distribution is a source of species differentiation and evolution (Raskina et al., 2008). In *Nigella*, the number of 45 rDNA loci varied from three to ten. However, no positive correlation between the loci number and genome size was observed. While the three larger genomes (*N. sativa*, *N. damascena* and *N. orientalis*) all showed only three loci, the highest number was found in *N. hispanica* which has a rather small genome. Also in diploid lineages of Brassicaceae (Hasterok et al., 2006), Cyperaceae (Da Silva et al., 2010), Iris (Martinez et al., 2010), and Rosaceae (Mishima et al., 2002) no positive correlation exists between the number of rDNA arrays and the number of chromosomes or genome size.

The presence of additional 45S rDNA loci in *N. damascena* (4 loci), *N. arvensis* (4 loci) and *N. hispanica* (10 loci) compared with *N. sativa*, *N. orientalis, N. integrifolia* and *N. bucharica* (all 3 loci) may be due to independent formation of a separate 45S rDNA array after the divergence of these species. *N. hispanica* also showed hemizygosity for one 45S rDNA site. rDNA site hemizygosity has been reported in other genera such as *Anacyclus* (Rosato et al., 2017), *Vicia* (Li et al., 2001), *Chrysanthemum* (He et al., 2021) and *Lilium* (Wang et al., 2012). In most cases such a heterozygosity is related to hybridization events. The reason for the detected hemizygous locus in *Nigella* remains to be elucidated. Similarly, it is not clear if this heterozygosity is only occurring in the investigated genotype or if it is a general feature of *N. hispanica*.

Overall, 45S rDNA composed about 0.38, 0.72, and 0.65% of the *N. damascena*, *N. sativa* and *N. bucharica* genomes, respectively (Table 2). The relative amount and size of rDNA units in the nuclear genome can be highly variable, and the rDNA copy number can vary between 150 to 26,048 copies in plants (Prokopowich et al., 2003; Wicke et al., 2011).

In *Nigella*, the majority (47.91% in *N. sativa*, 39.47% in *N. damascena* and 51.25% in *N. bucharica*) of the repeats are retrotransposons (class I transposons), while DNA transposons (class II transposons) contributed to only 1.13, 0.62, and 1.19% of the genomes. Although the three *Nigella* species possess rather large genomes of about 7.4 to 12.4 Gb/C, only one to four satellite repeats were found in these species, and no correlation was found between the number of satellites and the size of the genome in the studied *Nigella* species. Interestingly, *N. bucharica* and *N. integrifolia* with smaller genome sizes contained a higher number of satellite sequences than the larger genome of *N. damascena*. The most abundant satellites in the three species were found in (peri)centromeric position on all chromosomes.

Ns-CL6 is a retrotransposon distributed over all chromosomes of *N. sativa* although with a reduced density at distal regions. The reduced frequency of Ns-CL6 at chromosome ends could be explained by the potential enrichment of coding sequences in this region. In many plant species, especially well investigated in cereals, the terminal and subterminal chromosomal regions are often enriched in coding sequences^1^.

The rDNA probes alone or in combination with chromosome-specific satellite sequences are useful markers to identify individual chromosomes. While in *N. damascena*, *N. hispanica* and *N. orientalis* the 5S rDNA and 45S rDNA were sufficient to characterize the complete chromosome set, in *N. sativa* additionally NS-Sat2 was required. In *N. arvensis* 4 out of 6 and in *N. integrifolia* and *N. bucharica* 2 out of 7 chromosome pairs could be unequivocally identified by using the rDNA probes.

Sequence alignment indicated that the Ns-Sat1, Ns-Sat2 and Nd-Sat1 (peri)centromeric repeats are similar, suggesting they might share a common origin. However, the retained identity between Ns-Sat1 and Nd-Sat1 (78.8%, Supplementary Figure 2) was not enough for each of them to cross-hybridize on the other species, indicating their sequence divergence after specification. Ns-Sat1, Ns-Sat2 and Nd-Sat1 are all 178 bp long AT-rich satellite repeats. Due to their localization patterns and their length similarity with described centromeric satellites in other species such as *Arabidopsis* (Copenhaver et al., 1999), human (Choo et al., 1991) and the fish *Pungitius pungitius* (Varadharajan et al., 2019) it is tempting to speculate that these sequences indeed represent centromeric repeats of *Nigella*, although a functional proof is still missing. The 178 bp satellite unit is consistent with the 150–180 bp length DNA required to wrap around a single nucleosome (Henikoff et al., 2001). However, the (peri)centromeric satellites in *N. bucharica* and *N. integrifolia* (Nb-Sat3 and Nb-Sat4) have a deviating monomer length of only 135 and 39 bp.

Our molecular phylogeny using *rbc*L and ITS1-5.8S-ITS2 sequences grouped the seven *Nigella* species into three different clades, two groups with *x* = 6 and the third one with *x* = *7*. These results are in agreement with the morphological classifications reported earlier (Zohary, 1983: Yao et al., 2019). Significant variation observed in the sites and numbers of 45S rDNA loci might be involved in shaping *Nigella* karyotypes. The more asymmetric karyotype of the third group with additional telo- or subtelocentric chromosomes and the presence of terminal 45S rDNA sites in almost all telo- and subtelocentrics suggest that chromosomal rearrangements might play a role in changing the basic chromosome number (dysploidy) in the genus *Nigella*. DNA breakage and repair, rDNA mobility and Robertsonian fusions/fissions are suggested as the possible mechanisms during this process (Sramkó et al., 2019).

## Conclusion

Overall, our analyses based on the molecular phylogeny, DNA C-value analysis, genomic repeat composition and FISH-karyotyping shed light on the genome organization and evolution of seven *Nigella* species and supports a classification into three different groups of which two are closer to each other than the third one. The two phylogenetically closer groups (*N. sativa*, *N. arvensis* and *N. hispanica* and accordingly *N. damascena* and *N. orientalis*) share the same basic chromosome number (*x* = 6), and a similar karyotype formula. *N. integrifolia* and *N. bucharica*, in contrast, differ with *x* = 7 from the other five species. The repeatome analysis demonstrated that the genomes of *Nigella* species increased in size due to the preferential accumulation of Ty3_gypsy retroelements, especially of the Ty3_gypsy-Tekay lineage. In contrast, satellite repeats comprise only a small proportion of the *Nigella* genomes and are predominantly located at the (peri)centromeric regions. These sequences are only cross-hybridizing within the closely related species and support the proposed grouping. Surprisingly, despite the low total genome proportion of 5S and 45S rDNA, their diverse loci number and patterns on chromosomes of the analyzed species indicated the potential importance of rDNAs in driving the *Nigella* genome divergence and specification. Additionally, 5S and 45S rDNAs can be further applied as cytogenetic markers for chromosome discrimination and karyotype analysis in the genus *Nigella*.

## Data Availability Statement

The datasets presented in this study can be found in online repositories. The low coverage genomic DNA sequencing data of *N. sativa*, *N. damascena* and *N. bucharica* have been submitted to the Sequence Read Archive (SRA) database (https://www.ncbi.nlm.nih.gov/sra/) under accession number: PRJNA686272.

## Author Contributions

FO conducted the experiments and data analysis and assisted in the manuscript writing. GM conceived and designed the research and wrote the manuscript. Y-TK contributed to the data analysis, critical discussions, and manuscript revisions. JF contributed to the flow cytometry analysis and critical discussions. All authors contributed to the article and approved the submitted version.

## Conflict of Interest

The authors declare that the research was conducted in the absence of any commercial or financial relationships that could be construed as a potential conflict of interest.

## Publisher’s Note

All claims expressed in this article are solely those of the authors and do not necessarily represent those of their affiliated organizations, or those of the publisher, the editors and the reviewers. Any product that may be evaluated in this article, or claim that may be made by its manufacturer, is not guaranteed or endorsed by the publisher.

## Footnotes

1
https://plants.ensembl.org/index.html

## References

[B1] Abdolmalaki Z. Mirzaghaderi G. Mason A. S. Badaeva E. D. (2019). Molecular cytogenetic analysis reveals evolutionary relationships between polyploid *Aegilops* species. *Plant. Syst. Evol.* 305 459–475. 10.1007/s00606-019-01585-3

[B2] Abirached-Darmency M. Prado-Vivant E. Chelysheva L. Pouthier T. (2005). Variation in rDNA locus number and position among legume species and detection of 2 linked rDNA loci in the model *Medicago truncatula* by FISH. *Genome* 48 556–561. 10.1139/g05-015 16121252

[B3] Aboul-Maaty N. A.-F. Oraby H. A.-S. (2019). Extraction of high-quality genomic DNA from different plant orders applying a modified CTAB-based method. *Bull. Natl. Res. Cent.* 43:25. 10.1186/s42269-019-0066-1

[B4] Andrews S. (2010). *FastQC: A Quality Control Tool for High Throughput Sequence Data.* Cambridge: The Babraham Institute.

[B5] Appels R. Gerlach W. Dennis E. Swift H. Peacock W. (1980). Molecular and chromosomal organization of DNA sequences coding for the ribosomal RNAs in cereals. *Chromosoma* 78 293–311. 10.1007/s00239-004-0244-z 15983872

[B6] Atamuratova N. Mukhamatzanova R. Ch B. K. (2021). Honey significance of forest lands in south Uzbekistan. *IOP Conf. Ser. Earth Environ. Sci.* 775: 012013. 10.1088/1755-1315/775/1/012013

[B7] Bao W. Kojima K. K. Kohany O. (2015). Repbase update, a database of repetitive elements in eukaryotic genomes. *Mobile DNA* 6:11. 10.1186/s13100-015-0041-9 26045719 PMC4455052

[B8] Belyayev A. Josefiová J. Jandová M. Kalendar R. Krak K. Mandák B. (2019). Natural history of a satellite DNA family: from the ancestral genome component to species-specific sequences, concerted and non-concerted evolution. *Int. J. Mol. Sci.* 20:1201. 10.3390/ijms20051201 30857296 PMC6429384

[B9] Bennett M. D. Smith J. (1976). Nuclear DNA amounts in angiosperms. *Philos. Transac. R. Soc. Lond. B Biol. Sci.* 274 227–274. 10.1098/rstb.1976.0044 6977

[B10] Bennett M. D. Leitch I. J. Price H. J. Johnston J. S. (2003) Comparisons with *Caenorhabditis* (∼100 Mb) and Drosophila (∼175 Mb) using flow cytometry show genome size in Arabidopsis to be ∼157 Mb and thus ∼25% Larger than the Arabidopsis genome initiative estimate of ∼125 Mb. *Ann. Bot.* 91, 547–557. 10.1093/aob/mcg057 12646499 PMC4242247

[B11] Brown N. P. Leroy C. Sander C. (1998). MView: a web-compatible database search or multiple alignment viewer. *Bioinformatics* 14 380–381. 10.1093/bioinformatics/14.4.380 9632837

[B12] Choo K. Vissel B. Nagy A. Earle E. Kalitsis P. (1991). A survey of the genomic distribution of alpha satellite DNA on all the human chromosomes, and derivation of a new consensus sequence. *Nucleic Acids Res.* 19:1179. 10.1093/nar/19.6.1179 2030938 PMC333840

[B13] Copenhaver G. P. Nickel K. Kuromori T. Benito M.-I. Kaul S. Lin X. (1999). Genetic definition and sequence analysis of *Arabidopsis* centromeres. *Science* 286 2468–2474. 10.1126/science.286.5449.2468 10617454

[B14] Da Silva C. Quintas C. C. Vanzela A. L. (2010). Distribution of 45S and 5S rDNA sites in 23 species of Eleocharis (Cyperaceae). *Genetica* 138 951–957. 10.1007/s10709-010-9477-5 20680404

[B15] Datson P. Murray B. (2003). The use of in situ hybridisation to investigate plant chromosome diversity. *Plant Genome* 1:298Y318.

[B16] Dolezel J. Bartos J. Voglmayr H. Greilhuber J. (2003). Nuclear DNA content and genome size of trout and human. *Cytometry A* 51 127–8; author reply 129. 10.1002/cyto.a.10013 12541287

[B17] Dönmez A. S. Aydin Z. Uand Dönmez E. O. (2021). Taxonomic monograph of the tribe Nigelleae (Ranunculaceae): a group including ancient medicinal plants. *Turkish J. Bot.* 45 468–502. 10.3906/bot-2105-39

[B18] Elder J. F. Turner B. J. (1995). Concerted evolution of repetitive DNA sequences in eukaryotes. *Q. Rev. Biol.* 70 297–320. 10.1086/419073 7568673

[B19] Evans G. Rees H. Snell C. Sun S. (1972). The relationship between nuclear DNA amount and the duration of the mitotic cycle. *Chromosomes Today* 3 24–31.

[B20] Frello S. Heslop-Harrison J. (2000). Chromosomal variation in *Crocus vernus* Hill (Iridaceae) investigated by in situ hybridization of rDNA and a tandemly repeated sequence. *Ann. Bot.* 86 317–322.

[B21] Fu J. Zhang H. Guo F. Ma L. Wu J. Yue M. (2019). Identification and characterization of abundant repetitive sequences in *Allium cepa*. *Sci. Rep.* 9:16756. 10.1038/s41598-019-52995-9 31727905 PMC6856378

[B22] Garrido-Ramos M. (2017). Satellite DNA: an evolving topic. *Genes* 8:230. 10.3390/genes8090230 28926993 PMC5615363

[B23] Gerlach W. Bedbrook J. (1979). Cloning and characterization of ribosomal RNA genes from wheat and barley. *Nucleic Acids Res.* 7 1869–1885. 10.1093/nar/7.7.1869 537913 PMC342353

[B24] Gerlach W. Dyer T. (1980). Sequence organization of the repeating units in the nucleus of wheat which contain 5S rRNA genes. *Nucleic Acids Res.* 8 4851–4865. 10.1093/nar/8.21.4851 7443527 PMC324264

[B25] Ghosh A. Datta A. K. (2006). Karyotyping of *Nigella sativa* L.(black cumin) and *Nigella damascena* L.(love-in-a-mist) by image analyzing system. *Cytologia* 71 1–4. 10.1508/cytologia.71.1

[B26] Gilot-Delhalle J. Degraeve N. Moutschen J. (1976). Cytotaxonomic investigation of the genus Nigella (Helleboreae) with C-banding techniques. *Caryologia* 29 139–154. 10.1080/00087114.1976.10796656

[B27] Hasterok R. Wolny E. Hosiawa M. Kowalczyk M. Kulak-Ksiazczyk S. Ksiazczyk T. (2006). Comparative analysis of rDNA distribution in chromosomes of various species of Brassicaceae. *Ann. Bot.* 97 205–216. 10.1093/aob/mcj031 16357054 PMC2803362

[B28] He J. Lin S. Yu Z. Song A. Guan Z. Fang W. (2021). Identification of 5S and 45S rDNA sites in Chrysanthemum species by using oligonucleotide fluorescence in situ hybridization (Oligo-FISH). *Mol. Biol. Rep.* 48 21–31. 10.1007/s11033-020-06102-1 33454907

[B29] Heiss A. G. Kropf M. Sontag S. Weber A. (2011). Seed morphology of Nigella sl (Ranunculaceae): identification, diagnostic traits, and their potential phylogenetic relevance. *Int. J. Plant Sci.* 172 267–284. 10.1086/657676

[B30] Henikoff S. Ahmad K. Malik H. S. (2001). The centromere paradox: stable inheritance with rapidly evolving DNA. *Science* 293 1098–1102. 10.1126/science.1062939 11498581

[B31] IJdo J. W. Wells R. A. Baldini A. Reeders S. T. (1991). Improved telomere detection using a telomere repeat probe (TTAGGG)n generated by PCR. *Nucleic Acids Res.* 19:4780. 10.1093/nar/19.17.4780 1891373 PMC328734

[B32] Kuznetsova M. A. Chaban I. A. Sheval E. V. (2017). Visualization of chromosome condensation in plants with large chromosomes. *BMC Plant Biol.* 17:153. 10.1186/s12870-017-1102-7 PMC5596468 28899358

[B33] Leitch I. Johnston E. Pellicer J. Hidalgo O. Bennett M. (2019). *Plant DNA C-Values Database (Release 7.1, April 2019).* Available online at: https://cvalues.science.kew.org/

[B34] Levan A. Fredga K. Sandberg A. A. (1964). Nomenclature for centromeric position on chromosomes. *Hereditas* 52 201–220.

[B35] Levin D. A. Donald A. (2002). *The Role of Chromosomal Change in Plant Evolution.* Oxford: Oxford University Press.

[B36] Li R. Taylor S. Jenkins G. (2001). Unravelling the phytogeny of tetraploid vicia amoena (Fabaceae) and its diploid relatives using chromosomal landmarks. *Hereditas* 134 219–224. 10.1111/j.1601-5223.2001.00219.x 11833284

[B37] Madeira F. Park Y. M. Lee J. Buso N. Gur T. Madhusoodanan N. (2019). The EMBL-EBI search. *Nucleic Acids Res.* 47 W636–W641. 10.1093/nar/gkz268 30976793 PMC6602479

[B38] Malhotra S. (2012). “Nigella,” in *Handbook of Herbs and Spices*, Ed Peter K. V. (Amsterdam: Elsevier), 391–416. 10.1533/9780857095688.391

[B39] Martinez J. Vargas P. Luceno M. Cuadrado A. (2010). Evolution of Iris subgenus Xiphium based on chromosome numbers, FISH of nrDNA (5S, 45S) and trnL-trnF sequence analysis. *Plant Syst. Evol.* 289 223–235. 10.1007/s00606-010-0345-7

[B40] Maumus F. Quesneville H. (2016). Impact and insights from ancient repetitive elements in plant genomes. *Curr. Opin. Plant Biol.* 30 41–46. 10.1016/j.pbi.2016.01.003 26874965

[B41] Mehrotra S. Goyal V. (2014). Repetitive sequences in plant nuclear DNA: types, distribution, evolution and function. *Genomics Proteomics Bioinformatics* 12 164–171. 10.1016/j.gpb.2014.07.003 25132181 PMC4411372

[B42] Mahmoudi S. Mirzaghaderi G. (2021). Tools for drawing informative idiograms. *BioRxiv.* 10.1101/2021.09.29.459870 37335497

[B43] Mishima M. Ohmido N. Fukui K. Yahara T. (2002). Trends in site-number change of rDNA loci during polyploid evolution in Sanguisorba (Rosaceae). *Chromosoma* 110 550–558. 10.1007/s00412-001-0175-z 12068972

[B44] Mlinarec J. Papeš D. A. Besendorfer V. (2006). Ribosomal, telomeric and heterochromatin sequences localization in the karyotype of Anemone hortensis. *Bot. J. Linnean Soc.* 150 177–186. 10.1111/j.1095-8339.2006.00467.x

[B45] Mlinarec J. Šatović Z. Mihelj D. Malenica N. Besendorfer V. (2012). Cytogenetic and phylogenetic studies of diploid and polyploid members of tribe Anemoninae (Ranunculaceae). *Plant Biol.* 14 525–536. 10.1111/j.1438-8677.2011.00519.x 22188120

[B46] Mukai Y. Endo T. Gill B. (1991). Physical mapping of the 18S. 26S rRNA multigene family in common wheat: identification of a new locus. *Chromosoma* 100 71–78. 10.1007/BF00418239

[B47] Novák P. Ávila Robledillo L. Koblížková A. Vrbová I. Neumann P. Macas J. (2017). TAREAN: a computational tool for identification and characterization of satellite DNA from unassembled short reads. *Nucleic Acids Res.* 45:e111. 10.1093/nar/gkx257 28402514 PMC5499541

[B48] Novák P. Neumann P. Macas J. (2020). Global analysis of repetitive DNA from unassembled sequence reads using RepeatExplorer2. *Nat. Protoc.* 15 3745–3776. 10.1038/s41596-020-0400-y 33097925

[B49] Novak P. Neumann P. Pech J. Steinhaisl J. Macas J. (2013). RepeatExplorer: a Galaxy-based web server for genome-wide characterization of eukaryotic repetitive elements from next-generation sequence reads. *Bioinformatics* 29 792–793. 10.1093/bioinformatics/btt054 23376349

[B50] Olszewska M. J. Osiecka R. (1983). The relationship between 2 C DNA content, life cycle type, systematic position and the dynamics of DNA endoreplication in parenchyma nuclei during growth and differentiation of roots in some dicotyledonous herbaceous species. *Biochem. Physiol. Pflanzen* 178 581–599. 10.1016/S0015-3796(83)80073-0

[B51] Piégu B. Bire S. Arensburger P. Bigot Y. (2015). A survey of transposable element classification systems–a call for a fundamental update to meet the challenge of their diversity and complexity. *Mol. Phylogenet.* 86 90–109. 10.1016/j.ympev.2015.03.009 25797922

[B52] Prokopowich C. D. Gregory T. R. Crease T. J. (2003). The correlation between rDNA copy number and genome size in eukaryotes. *Genome* 46 48–50. 10.1139/g02-103 12669795

[B53] Raab-Straube E. von Hand R. Hörandl E. Nardi E. (2014). *Ranunculaceae. Euro+Med Plantbase.* Available at: http://ww2. bgbm. org/EuroPlusMed

[B54] Raskina O. Barber J. Nevo E. Belyayev A. (2008). Repetitive DNA and chromosomal rearrangements: speciation-related events in plant genomes. *Cytogenet. Genome Res.* 120 351–357. 10.1159/000121084 18504364

[B55] Roa F. Telles M. P. C. (2020). *idiogramFISH: Idiograms with Marks and Karyotype Indices. R-Package. Version 1.15.3.* Goiânia: Universidade Federal de Goiás.

[B56] Romero-Zarco C. (1986). A new method for estimating karyotype asymmetry. *Taxon* 35 526–530. 10.2307/1221906

[B57] Rosato M. Álvarez I. Nieto Feliner G. Rosselló J. A. (2017). High and uneven levels of 45S rDNA site-number variation across wild populations of a diploid plant genus (Anacyclus, Asteraceae). *PLoS One* 12:e0187131. 10.1371/journal.pone.0187131 PMC5663423 29088249

[B58] Saghai-Maroof M. A. Soliman K. M. Jorgensen R. A. Allard R. (1984). Ribosomal DNA spacer-length polymorphisms in barley: mendelian inheritance, chromosomal location, and population dynamics. *Proc. Natl. Acad. Sci. U.S.A.* 81 8014–8018. 10.1073/pnas.81.24.8014 6096873 PMC392284

[B59] Samoluk S. S. Robledo G. Bertioli D. Seijo J. G. (2017). Evolutionary dynamics of an at-rich satellite DNA and its contribution to karyotype differentiation in wild diploid *Arachis* species. *Mol. Genet. Genomics* 292 283–296. 10.1007/s00438-016-1271-3 27838847

[B60] Schubert I. (1984). Mobile nucleolus organizing regions (NORs) in Allium (Liliaceae s. lat.)?—Inferences from the specifity of silver staining. *Plant Syst. Evol.* 144 291–305. 10.1007/BF00984139

[B61] Shaker S. S. Mohammadi A. Shahli M. K. (2017). Cytological studies on some ecotypes of *Nigella sativa* L. in Iran. *Cytologia* 82 123–126. 10.1508/cytologia.82.123

[B62] Sramkó G. Laczkó L. Volkova P. A. Bateman R. M. Mlinarec J. (2019). Evolutionary history of the Pasque-flowers (Pulsatilla, Ranunculaceae): molecular phylogenetics, systematics and rDNA evolution. *Mol. Phylogenet. Evol.* 135 45–61. 10.1016/j.ympev.2019.02.015 30831271

[B63] Stebbins G. L. (1971). *Chromosomal Evolution in Higher Plants.* London: Edward Arnold (Publishers) Ltd.

[B64] Tagashira N. Kondo K. (2001). Chromosome phylogeny of Zamia and Ceratozamia by means of Robertsonian changes detected by fluorescence in situ hybridization (FISH) technique of rDNA. *Plant Syst. Evol.* 227 145–155. 10.1007/s006060170045

[B65] Tamura K. Stecher G. Kumar S. (2021). MEGA11: molecular evolutionary genetics analysis version 11. *Mol. Biol. Evol.* 38 3022–3027. 10.1093/molbev/msab120 33892491 PMC8233496

[B66] Tillich M. Lehwark P. Pellizzer T. Ulbricht-Jones E. S. Fischer A. Bock R. (2017). GeSeq – versatile and accurate annotation of organelle genomes. *Nucleic Acids Res.* 45 W6–W11. 10.1093/nar/gkx391 28486635 PMC5570176

[B67] Tutin T. G. (1964). “Nigella,” in *Flora Europaea1*, ed. Tutin T. G. Heywood V. H. Burges N. A. Valentine D. H. Walters S. M. Webb D. A. (Cambridge: University Press), 209–210.

[B68] Ugarkovic D. Plohl M. (2002). Variation in satellite DNA profiles-causes and effects. *EMBO* 2 5955–5959. 10.1093/emboj/cdf612 12426367 PMC137204

[B69] Untergasser A. Cutcutache I. Koressaar T. Ye J. Faircloth B. C. Remm M. (2012). Primer3—new capabilities and interfaces. *Nucleic Acids Res.* 40:e115. 10.1093/nar/gks596 22730293 PMC3424584

[B70] Varadharajan S. Rastas P. Löytynoja A. Matschiner M. Calboli F. C. F. Guo B. (2019). Genome sequencing of the nine-spined stickleback (*Pungitius pungitius*) provides insights into chromosome evolution. *Genome Biol. Evol.* 11 3291–3308. 10.1101/741751 31687752 PMC7145574

[B71] Wang X. Xie S. Zhang Y. Niu L. (2012). Chromosome analysis and mapping of ribosomal genes by fluorescence in situ hybridization (FISH) in four endemic lily species (Lilium) in Qinling Mountians, China. *Pak. J. Bot* 44 1319–1323.

[B72] Wicke S. Costa A. Muñoz J. Quandt D. (2011). Restless 5S: the re-arrangement(s) and evolution of the nuclear ribosomal DNA in land plants. *Mol. Phylogenet. Evol.* 61 321–332. 10.1016/j.ympev.2011.06.023 21757016

[B73] Wicker T. Sabot F. Hua-Van A. Bennetzen J. L. Capy P. Chalhoub B. (2007). A unified classification system for eukaryotic transposable elements. *Nat. Rev. Genet.* 8:973. 10.1038/nrg2165 17984973

[B74] Yao X. Zhang W. Duan X. Yuan Y. Zhang R. Shan H. (2019). The making of elaborate petals in Nigella through developmental repatterning. *N. Phytol.* 223 385–396. 10.1111/nph.15799 30889278

[B75] Zohary M. (1983). The genus *Nigella* (Ranunculaceae) — a taxonomic revision. *Plant Syst. Evol.* 142 71–105. 10.1007/BF00989605

[B76] Zoldos V. Papes D. Cerbah M. Panaud O. Besendorfer V. Siljak-Yakovlev S. (1999). Molecular-cytogenetic studies of ribosomal genes and heterochromatin reveal conserved genome organization among 11 Quercus species. *Theoret. Appl. Genet.* 99 969–977. 10.1007/s001220051404

