# Supplementary material for: Variation in the Number and Position of rDNA Loci Contributes to the Diversification and Speciation in Nigella (Ranunculaceae)
Source: Front Plant Sci. 2022 Jun 9;13:917310. doi: 10.3389/fpls.2022.917310 (PMC9261981; doi:10.3389/fpls.2022.917310)
Supplement: Supplementary file 1 [file Data_Sheet_1.docx]

Article title:

**Variation in number and position of rDNA loci is one of the driving forces in the diversification and speciation in *Nigella* (Ranunculaceae)**

Fatemeh Orooji^1^, Ghader Mirzaghaderi^1*^, Yi-Tzu Kuo^2^, Jörg Fuchs^2^

^1^ Department of Agronomy and Plant Breeding, Faculty of Agriculture, University of Kurdistan, Postal code 66177–15175, Sanandaj, Iran

^2^ Leibniz Institute of Plant Genetics and Crop Plant Research (IPK), 06466 Gatersleben, Stadt Seeland, Germany

^*^ Corresponding author (Email: gh.mirzaghaderi@uok.ac.ir)

**Supplementary figures**

**Figure S1.** Worldwide distribution of Nigella species

**References**

Raab-Straube, E. von, Hand, R., Hörandl, E. and Nardi, E. (2014) Ranunculaceae. – In: Euro+Med Plantbase.

Zohary, M. (1983) The genus *Nigella* (Ranunculaceae) — a taxonomic revision. Pl Syst Evol 142, 71–105.

Zohary, M. (1966) Flora Palaestina1, 190–195. — Jerusalem: Israel Acad. Sciences and Humanities.

Tutin, T. G. (1964) *Nigella*. — In Tutin, T. G., & al. (Eds.): Flora Europaea1, 209–210. — Cambridge: Univ. Press.


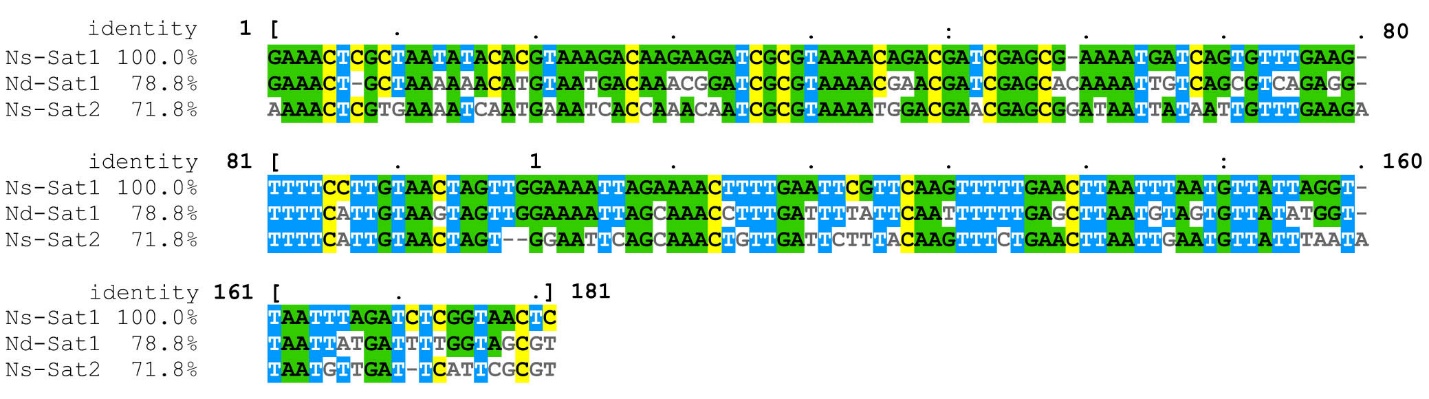


**Figure S2.** Alignment of the 178 bp, AT-rich (68% for Ns-Sat1) centromeric repeat monomers in *N. sativa* (Ns-Sat1 and Ns-Sat2) and *N. damascena* (Nd-Sat1).


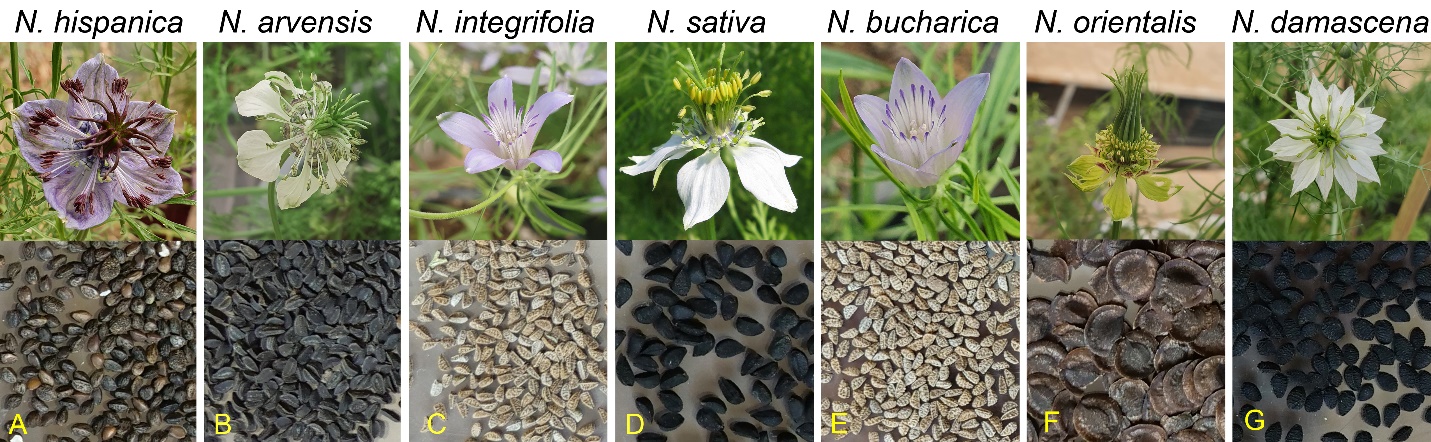


**Figure S3.** Flower and seed morphology and rDNA loci distribution of the studied species
